# Zerumbone Induces Apoptosis in Breast Cancer Cells by Targeting αvβ3 Integrin upon Co-Administration with TP5-iRGD Peptide

**DOI:** 10.3390/molecules24142554

**Published:** 2019-07-13

**Authors:** Eltayeb E. M. Eid, Abdulrahman S. Alanazi, Sanaz Koosha, Alian A. Alrasheedy, Faizul Azam, Ismail M. Taban, Habibullah Khalilullah, Mothanna Sadiq Al-Qubaisi, Mohammed A. Alshawsh

**Affiliations:** 1 Department of Pharmaceutical Chemistry and Pharmacognosy, Unaizah College of Pharmacy, Qassim University, 51911 Unaizah, Saudi Arabia; 2 College of Pharmacy, Hail University, Hail, Saudi Arabia; 3 Department of Pharmacology, Faculty of Medicine, University of Malaya, 50603 Kuala Lumpur, Malaysia; 4 School of Biosciences, Cardiff University, Cardiff CF10 3AT, UK; 5 Institute of Bioscience, University Putra Malaysia, 43400 UPM Serdang, Malaysia

**Keywords:** integrin, zerumbone, TP5-iRGD peptide, breast cancer

## Abstract

Cell-penetrating peptides (CPPs) are highly promising tools to deliver therapeutic molecules into tumours. αVβ3 integrins are cell–matrix adhesion receptors, and are considered as an attractive target for anticancer therapies owing to their roles in the process of metastasis and angiogenesis. Therefore, this study aims to assess the effect of co-administration of zerumbone (ZER) and ZERencapsulated in hydroxypropyl-β-cyclodextrin with TP5-iRGD peptide towards cell cytotoxicity, apoptosis induction, and proliferation of normal and cancerous breast cells utilizing in vitro assays, as well as to study the molecular docking of ZER in complex with TP5-iRGD peptide. Cell viability assay findings indicated that ZER and ZERencapsulated in hydroxypropyl-β-cyclodextrin (ZER-HPβCD) inhibited the growth of estrogen receptor positivebreast cancer cells (ER^+^ MCF-7) at 72 h treatment with an inhibitory concentration (IC)_50_ of 7.51 ± 0.2 and 5.08 ± 0.2 µg/mL, respectively, and inhibited the growth of triple negative breast cancer cells (MDA-MB-231) with an IC_50_ of 14.96 ± 1.52 µg/mL and 12.18 ± 0.7 µg/mL, respectively. On the other hand, TP5-iRGD peptide showed no significant cytotoxicity on both cancer and normal cells. Interestingly, co-administration of TP5-iRGD peptide in MCF-7 cells reduced the IC_50_ of ZER from 7.51 ± 0.2 µg/mL to 3.13 ± 0.7 µg/mL and reduced the IC_50_ of ZER-HPβCD from 5.08 ± 0.2 µg/mL to 0.49 ± 0.004 µg/mL, indicating that the co-administration enhances the potency and increases the efficacy of ZER and ZER-HPβCD compounds. Acridine orange (AO)/propidium iodide (PI) staining under fluorescence microscopy showed evidence of early apoptosis after 72 h from the co-administration of ZER or ZER-HPβCD with TP5-iRGD peptide in MCF-7 breast cancer cells. The findings of the computational modelling experiment provide novel insights into the ZER interaction with integrin αvβ3 in the presence of TP5-iRGD, and this could explain why ZER has better antitumor activities when co-administered with TP5-iRGD peptide.

## 1. Introduction

Breast cancer, with an estimated 2.1 million newly diagnosed cases in 2018, is considered as the most prevalent cancer, eliciting a high mortality rate among women worldwide [1]. Therapeutic armamentarium of this ailment relies upon chemotherapy, which is associated with a number of drawbacks, including the low concentration of chemotherapeutic drug at the tumor site, and stern adverse effects due to chemotherapeutic drugs not distinctively targeting tumors and always distressing normal cells/tissues [2]. Poor penetrability of anticancer drugs into tumors is considered as an important factor limiting their efficacy. In solid tumors, many antineoplastic drugs invade only three to five cell diameters deep from the blood vessels, leading to reduced efficacy as well as the development of drug resistance [3,4]. Furthermore, acquired resistance to chemotherapy thwarts the efficient treatment of the patients. Owing to these limitations, improving the efficacy and permeability of chemotherapeutic agents is an urgent requirement [5,6]. Several therapeutic options are available for the treatment of cancers, including breast cancer; however, none of them completely suppress the tumour metastases or disease progression. Thus, there is a definite need for new therapeutic development approaches, such as peptide-drug conjugates for cancer targeting therapy [7].

αVβ3 integrin, a heterodimeric transmembrane receptor, consists of one α- and one β-subunit. These proteins are involved in the regulation of numerous biological processes, such as extracellular matrix assembly, cell adhesion, cell migration, and function as scaffolds for various signal transduction pathways [8,9]. Therapeutic targeting of integrins for anticancer drug development has gained profound interest among researchers nowadays [10].

Natural products have always played a pivotal role in various drug discovery and development programs by serving either as sources of pharmaceutical drugs in the form of isolated plant compounds, as sources of precursors to drugs, or as sources of compounds that have served as models for synthetic or semisynthetic drugs [11]. A substantial number of commercialized medicines have been developed from natural sources in the field of oncology. For example, vinca alkaloids from *Catharanthus roseus*, and terpene paclitaxel from *Taxus baccata*, are among successful anticancer drugs originally derived from plants, narrating the conspicuous accomplishments of natural products in modern medicine [12].

Zerumbone (ZER, M.wt. 218.34 g/mol) is a sesquiterpene compound derived from the rhizomes of the wild ginger, *Zingiber zerumbet* smith. The anti-breast cancer properties of zerumbone have been revealed by several studies [13,14,15,16]. ZER suppressesMCF-7 and MDA-MB-23 breast cancer cells via inducing apoptosis and shows robust activation of both Bax and Bak [15]. In addition, administration of ZER showed that in association with apoptosis induction, cell proliferation has been suppressed by inhibition of Ki-67 protein [15]. Kim et al. claimed that ZER inhibits epidermal growth factor (EGF)-induced CD44 through suppression of the signal transducer and activator of transcription 3 (STAT-3) signalling pathway in SKBR3 human breast cancer cells [17]. ZER has several desirable pharmacological activities ranging from anti-inflammatory to anti-cancer properties; however, the therapeutic efficiency and potency of ZER and its mechanism towards breast cancer are unknown, although the initial development of ZER is well reported [13,18]. Although ZER has several desirable pharmacologic properties against cancer, therapeutic efficiency of this compound is limited because of its poor penetration into cancer cells, which can lead to drug resistance. Moreover, ZER has very poor water solubility, which affects its therapeutic applications. Hence, ZER can be encapsulated in HPβCD to increase its hydrophilicity [13].

Cell-penetrating peptides (CPPs) have shown potential to facilitate the cellular delivery and to increase the cellular uptake of different therapeutic molecules such as drugs, DNAs, and proteins [19,20,21]. In general, CPPs are non-toxic short peptides consistingof 5–40 amino acids, and are able to penetrate tumour cells mediated by various mechanisms, including endocytosis and direct membrane penetration [22,23]. Many studies have reported that CPPs are a highly promising strategy for cancer therapy through the delivery of covalently or non-covalently conjugated molecules into tumour cells, and thereby increase the efficacy of the treatment [24,25,26,27,28]. During the last decade, different CPPs have been reported for in vitro and in vivo delivery of anticancer therapeutic molecules [29,30]. However, it is very interesting to design short CPPs that possess active and useful cell penetrating properties, specific targeting capacity, and good stability in biological fluids. Sugahara et al. reported that co-administration of iRGDtumour-penetrating peptide with chemotherapeutic agents can carry the drugs deeper to the tumour tissue, and enhances the efficacy of treatment [31]. However, the poor selectivity of CPPs is the main drawback that limits its uses. Therefore, it is very interesting to design short selective CPPs that possess an active and useful cell penetrating property, specific targeting capacity, and good stability in a biological environment [32,33]. Another recent study demonstrated thatiRGD peptide has increased the penetration of Nab-paclitaxel in BT474 breast cancer cells [34]. In another study, Lao et al. exploited conjugated iRGD with the TP5 C-terminus to yield a tumor-penetrating peptide (TP5-iRGD) in order to increase both the tumor-homing and antitumor effects of TP5 [35]. Furthermore, the co-administration of doxorubicin with CPPs increased the efficiency of doxorubicin [36,37]. Recently, it has also been reported that co-administration of iRGD with multistage-responsive nanoparticles enhances drug delivery, efficiency, and penetration of the cancer tumour [38,39]. Therefore, this study aims to investigate the co-administration of natural ZER and ZER-HPβCD complex with TP5-iRGD peptide against breast cancer cells lines. 

## 2. Materials and Method

### 2.1. Compounds and Peptide

**Figure 1:**
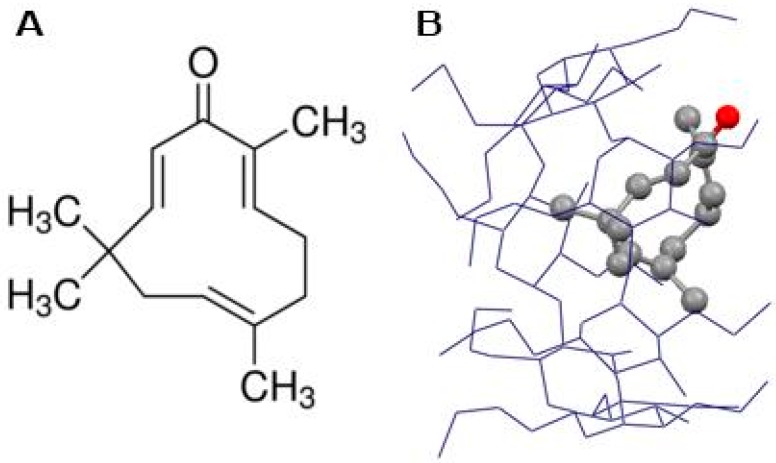
Chemical structure of zerumbone (**A**) and zerumbone encapsulated in hydroxypropyl-β-cyclodextrin (ZER -HPβCD) (**B**).

Zerumbone (ZER) and hydroxypropyl-β-cyclodextrin (HPβCD) were purchased from Sigma-Aldrich (St. Louis, MO, USA) with purity >99%. TP5-iRGDpeptide (H-RKDVYCRGDKGPDC-NH2) (cyclic) was synthesized and purified byPepscan Presto (Lelystad, Netherlands). The TP5-iRGDpurity was assessed by ultra-performance liquid chromatography (UPLC) and mass spectrometry, the purity was 99.4%, and was delivered as a lyophilized powder to be used in in vitro experimental protocols.ZER was encapsulated in HPβCD to increase its hydrophilicity and the ZER-HPβCD mixture (Figure 1B) was prepared as described previously in our publication [13]. 

### 2.2. Cell Lines

Human breast cancer cells (MCF-7 and MDA-MB-231) and human normal fibroblast cells (Hs27) were obtained from the American Type Culture Collection, (ATCC, Manassas, VA, USA). MCF-7 breast cancer cells are estrogen receptor positive (ER^+^), while MDA-MB-231 breast cancer cells are triple negative. The Hs27 cell line resembles epithelial cells morphologically. Stock cells were routinely maintained in Dulbecco’s modified eagle’s medium (DMEM); supplemented with 10% fetal bovine serum, penicillin (100 U/mL), and streptomycin (100 g/mL); and incubated in a humidified 5% CO_2_ at 37 °C. 

### 2.3. MTT Cell Viability Assay for the Individual Compounds

Cell viability assay for the MCF-7, MDA-MB-231 (breast cancer cells), and Hs27 (normal fibroblast) cells was carried out using the 3-(4,5-dimethylthiazol-2-yl)-2,5-diphenyltetrazolium bromide (MTT) assay [40]. MTT assay is based on the reduction of yellow tetrazolium MTT into purple formazan crystals by living cells, which reflects mitochondrial activity. This assay is widely used to measure the in vitro cytotoxic effects of drugs on cell lines.

Confluent cells were seeded in 96-well culture plates at a density of 5000 cells/well. After 24 h, cells were treated with sixserial dilutions (100, 50, 25, 12.5, 6.25, and 3.125 µg/mL) of ZER and ZER-HPβCD, and incubated at 37 °C for 24, 48, and 72 h. Cells also were treated withTP5-iRGD peptide at concentrations of 1000, 500, 250, 125, 62.5, and 31.25 µg/mL for 48 and 72 h. Control-untreated cells were incubated with culture medium and vehicle. Then, 20 µL of MTT (1 mg/mL in phosphate-buffered saline (PBS) was added to each well, and the microplates were further incubated at the same conditions for four hours. After incubation, the solution in each well was discarded, and 100 µL dimethyl sulfoxide (DMSO) was added into the wells to solubilize the produced formazan. The optical density was measured at 570 nm spectral wavelength using a microliter plate reader. The concentration of compounds that inhibit 50% of cell growth was expressed as the median inhibitory concentration (IC_50_) value, which was calculated using Graph Pad Prism (La Jolla, CA, USA), version 6.07 for windows. Cell viability assay was performed in triplicate and the IC_50_ of ZER and ZER-HPβCD compounds were used for the co-administration MTT assay.

### 2.4. MTT Cell Viability Assay for the Co-Administration

MCF-7 and MDA-MB-231 cells were treated with a combination of the IC_50_ of ZER or ZER -HPβCD with TP5-iRGD peptide, and cell viability was measured using MTT assay as described above. Approximately 5000 cells/well were seeded and incubated for 24 h. The IC_50_ of ZER or ZER -HPβCD in µg/mL was co-administered with TP5-iRGD peptide at ratios of 1:10, 1:20, and 1:30 and incubated for 1 h and loaded into respective wells in triplicate. After treatment, the plates were incubated for 72 h and the IC_50_ of each co-administration was calculated. The co-administration of ZER or ZER-HPβCD with TP5-iRGD peptide at a ratio of 1:10 was more effective at killing MCF-7 cancer cells than ZER and ZER-HPβCD alone, which led us to use the IC_50_ of this co-administration for the downstream assays.

### 2.5. Dual-Fluorescence for Live/Dead Nucleated Cell Assay

The viability state of the MCF-7 cells at IC_50_ of the co-administered compounds was further investigated using double fluorescent dye staining method acridine orange (AO) and propidium iodide (PI) and measured via fluorescence microscopy. AO dye stains all live and dead nucleated cells and generates green fluorescence, while PI dye only stains dead nucleated cells with red fluorescence. After MCF-7 cells were treated with ZER-TP5-iRGD or ZER-HPβCD-TP5-iRGD compounds and incubated for 72 h, the cells were detached and washed twice using PBS to remove excess media. Then, 10 µL of fluorescent dyes containing AO (10 µg/mL) and PI (10 µg/mL) was added into the wells in equal volumes. Freshly stained cell suspension was dropped onto a glass slide and covered by coverslip. The slides were then observed under an Olympus BX50 fluorescencemicroscope (Tokyo, Japan) using different wavelengths, and the photos were captured [41].

### 2.6. Molecular Docking

ZER structure was taken from PubChem and drawn using ChemBioDraw Ultra 12.0 (CambridgeSoft, Cambridge, UK) and converted to their three-dimensional structure in ChemBio3D Ultra 12.0, energy minimized by the PM3 method using the MOPAC Ultra 2009 program (http://OpenMOPAC.net). The prepared ligand was used as input file for AutoDock 4.2 in the next step. Lamarckian genetic algorithm method was employed for docking simulations [42]. The standard docking procedure was used for a rigid protein and a flexible ligand whose torsion angles were identified (for 100 independent runs). A grid of 60, 60, and 60 points in x, y, and z directions was built with a grid spacing of 0.375 Å, and a distance-dependent function of the dielectric constant was used for the calculation of the energetic map. The default settings were used for all other parameters. At the end of docking, the best poses were analyzed for hydrogen bonding/π–π interactions, and root mean square deviation (RMSD) was calculated using Discovery Studio Visualizer 2.5 program (Accelrys Software Inc., San Diego, CA, USA). The inhibition constant (*K*_i_) for each ligand was calculated from the estimated free energy of ligand binding (Δ*G*_binding_, kcal/mol).

### 2.7. Molecular Dynamics Simulation

Molecular dynamics (MD) simulation using Desmond [43,44] was performed to analyze the stability of the docked ZER-HPβCD-TP5-iRGD complex, as well as the pattern of interaction during the simulated trajectory. Minimum energy conformation of ZER in complex with αvβ3 integrin was obtained from the molecular docking study and kept in an orthorhombic box with 53654 water molecules. Simple point charge (SPC) solvent model and the optimized potential for liquid simulations (OPLS3) force field [45] were employed for simulation. In order to neutralize the complex, salt of 0.15 M concentration was used to append a suitable number of Na^+^/Cl^−^ counter-ions to the system. Temperature and pressure were adjusted to 300 K and 1.01325 bar, respectively, by employing isothermal-isobaric (NPT) ensemble class. A Nose–Hoover thermostat [46] and Martyna–Tobias–Klein [47] approaches were implemented to maintain the temperature and the pressure of the systems, respectively. Simulation time was set to 20 ns and the coordinates were saved for every 5 ps. A cut-off radius of 9.0 Å was applied for short-range van der Waals and Coulomb interactions. The reference system propagator algorithms (RESPA) integrator was exercised with a time step of 2.0 fs [48] for the overall simulations. Default protocol of the Desmond was set for the minimization and equilibration of the system.

### 2.8. Statistical Analysis

All data were expressed as mean ± standard deviation (SD) of three replicates. Statistical analyses were performed using SPSS for windows, version 21 (SPSS Inc., Chicago, IL, USA).

## 3. Results

### 3.1. MTT Assay of Individual Compounds

**Figure 2:**
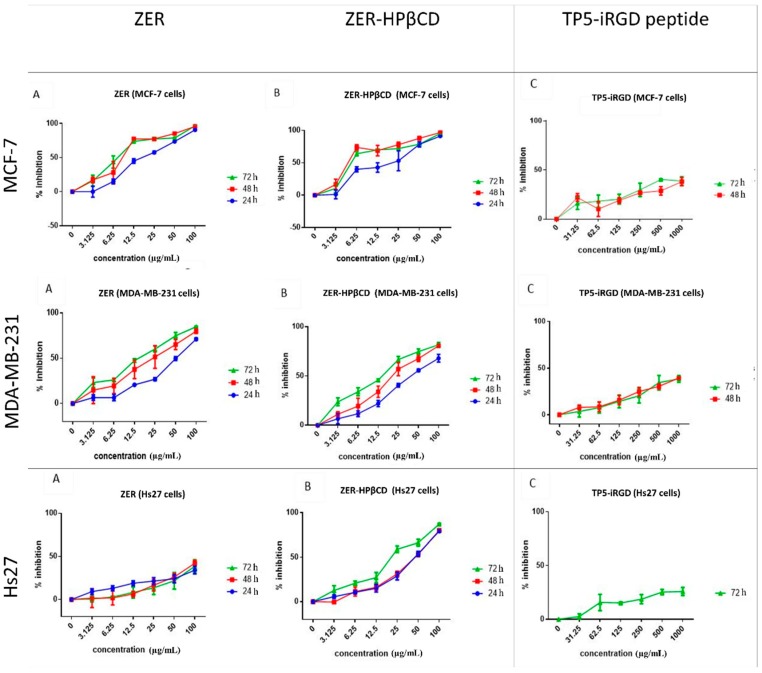
Percentage of cell inhibition (3-(4,5-dimethylthiazol-2-yl)-2,5-diphenyltetrazolium bromide(MTT) assay) of MCF-7, MDA-MB-231, and Hs27 cells treated with zerumbone (ZER) (**A**), ZER-hydroxypropyl-β-cyclodextrin(HPβCD) (**B**), and TP5-iRGD peptide (**C**).

The cytotoxicity of ZER, ZER-HPβCD, and TP5-iRGD peptide was investigated in MCF-7, MDA-MB-231 (breast cancer cells), and Hs27 (normal fibroblast cells) at different time points. Findings indicate that at 72 h, ZER and ZER-HPβCD exhibited the best cytotoxicity effect against MCF-7 breast cancer cells with an IC_50_ of 7.51 ± 0.2 µg/mL and 5.08 ± 0.2 µg/mL, respectively. On the other hand, ZER and ZER-HPβCD demonstrated less cytotoxicity against MDA-MB-231 breast cancer cells with an IC_50_ of 14.96 ± 1.5 µg/mL and 12.18 ± 0.7 µg/mL, respectively. Therefore the IC_50_ of compounds after 72 h of treatment was chosen for the co-administration assessment. On the other hand, TP5-iRGD peptide has no cytotoxic effect against both breast cancer and normal cells. Besides, ZER-HPβCD showed slight cytotoxicity against Hs27 normal cells with IC_50_ of21.45 ± 1.3 µg/mL after 72 h treatment, while ZER has a low cytotoxicity in Hs27 cells with IC_50_ > 100 µg/mL (Figure 2 and Table 1).

### 3.2. MTT Assay of the Co-Administration

**Figure 3:**
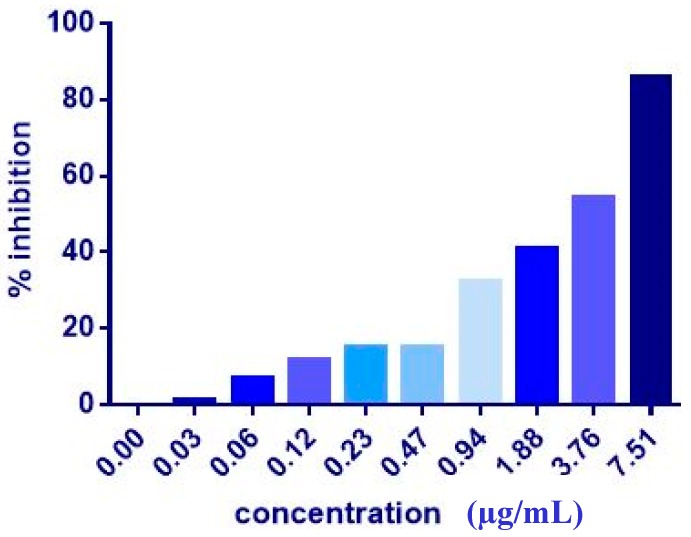
Of cell inhibition (MTT assay) of MCF-7 cells treated with ZER-TP5-iRGD for 72 h.

**Figure 4:**
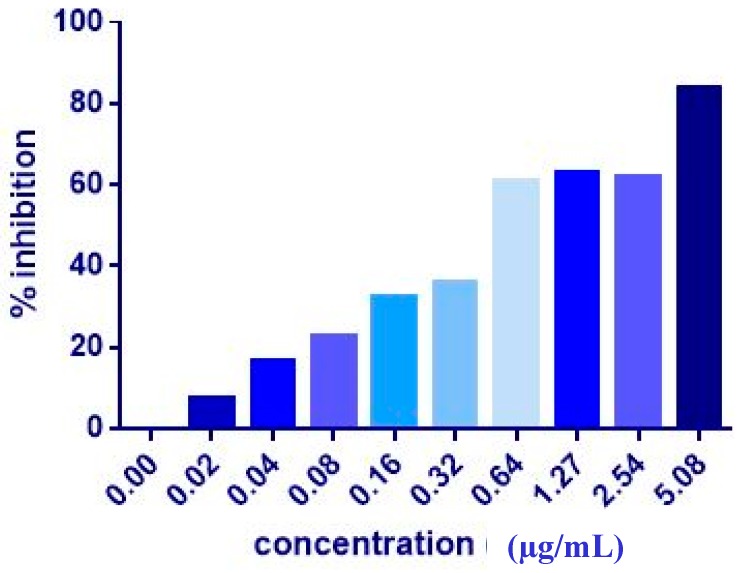
Of cell inhibition (MTT assay) of MCF-7 cells treated with ZER-HPβCD-TP5-iRGD for 72 h.

The co-administration of ZER or ZER-HPβCD with TP5-iRGD peptide at three different ratios (1:10, 1:20, and 1:30) wasevaluated after 72 h treatment against MCF-7 and MDA-MB-231 breast cancer cells. Co-administration of IC_50_ of ZER or ZER-HPβCD with 10× (10 times) TP5-iRGD showed the highest inhibition in MCF-7 cells (91.4 and 90.4%), respectively. However, the combination demonstrated non-significant inhibition against MDA-MB-231 cells compared with the ZER and ZER-HPβCD alone (Figure 3, Figure 4 and Table 2). The obtained results showed that co-administration treatment with TP5-iRGD in MCF-7 cells reduced the IC_50_ of ZER from 7.51 ± 0.2 µg/mL to 3.13 ± 0.7 µg/mL and reduced the IC_50_ of ZER-HPβCD from 5.08 ± 0.2 µg/mL to 0.49 ± 0.004 µg/mL (Table 3). Therefore, the IC_50_ of ZER or ZER-HPβCD with 10× of TP5-iRGD peptide were used for the downstream assay. 

### 3.3. Apoptosis Detection by Dual Staining

**Figure 5:**
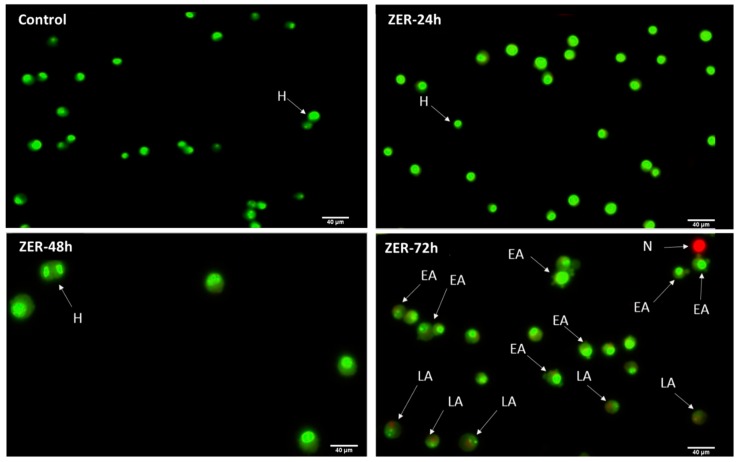
Acridine orange (AO)/propidium iodide (PI) assay for MCF-7 cells treated with the co-administration of zerumbone and TP5-iRGD peptide at three different time points of 24, 48, and 72 h. H: healthy, EA: early apoptosis, LA: late apoptosis, N: necrosis.

**Figure 6:**
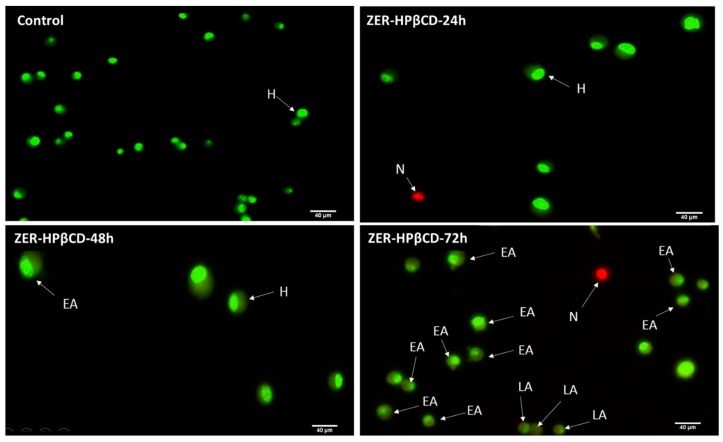
AO/PI assay for MCF-7 cells treated with the co-administration of ZER-HPβCD and TP5-iRGD peptide at three different time points of 24, 48 and 72h. H: healthy, EA: early apoptosis, LA: late apoptosis, N: necrosis.

The AO/PI assay finding indicates that in the presence of a combination of ZER at 3.13 µg/mL and 10× TP5-iRGD peptide, MCF-7 cells underwent apoptosis after 72 h. The same results were obtained when MCF-7 was exposed to 0.49 µg/mL of ZER-HPβCD co-administered with TP5-iRGD peptide. Figure 5 and Figure 6 show that co-administration of ZER or ZER-HPβCD with TP5-iRGD peptide for 72 h resulted in morphological changes in breast cancer cells such as membrane blebbing, loss of nuclear architecture, and apoptotic bodies, which indicate early apoptosis. Meanwhile, treatment for 24 and 48 h showed less nuclear changes occurred. 

### 3.4. Molecular Docking

**Figure 7:**
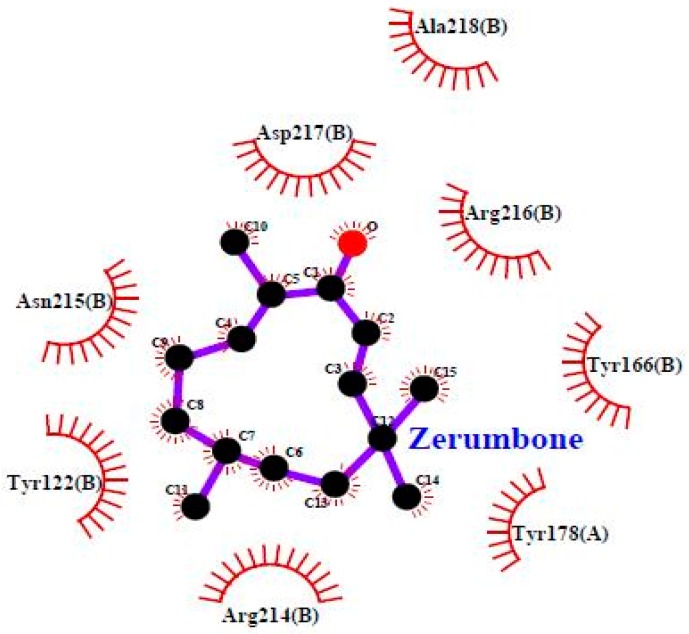
Zerumbone in complex with integrin αvβ3 (Protein Data Bank (PDB) code: 1L5G).

To investigate the mechanism by which TP5-iRGD peptide increases the anticancer activity of ZER, we used computational modelling to explore its binding pattern. The binding mode of ZER in complex with integrin αvβ3/TP5-iRGD in silico is presented in Figure 7. 

ZER was docked into the integrin αvβ3 exhibiting binding energy of −6.77 kcal/mol, whereas when ZER was docked in the presence of TP5-iRGD peptide, the observed binding energy decreased to −8.13 kcal/mol. Similarly, very few residues were involved in the binding interaction with ZER when docked alone (Table 4). However, robust interaction was observed when ZER was docked in the presence of TP5-iRGD (Figure 7). The iRGD component of TP5-iRGD peptide was slightly embedded in the head interface between the αv and β3 subunits, similar to the structure described in 1L5G. The main contact points between integrin and iRGD include Tyr178 in the αv subunit and Tyr122, Tyr166, Tyr178, Arg214, Asn215, Arg216, Asp217, and Ala-218 in the β3 subunit. The findings from our computational modelling experiment provide novel insights into the ZER interaction with integrin αvβ3 in the presence of TP5-iRGD, and help explain why ZER has better antitumor activities when co-administered with TP5-iRGD.

### 3.5. Molecular Dynamics Simulation Studies of ZER in Complex with TP5-iRGD/Integrin αvβ3

**Figure 8:**
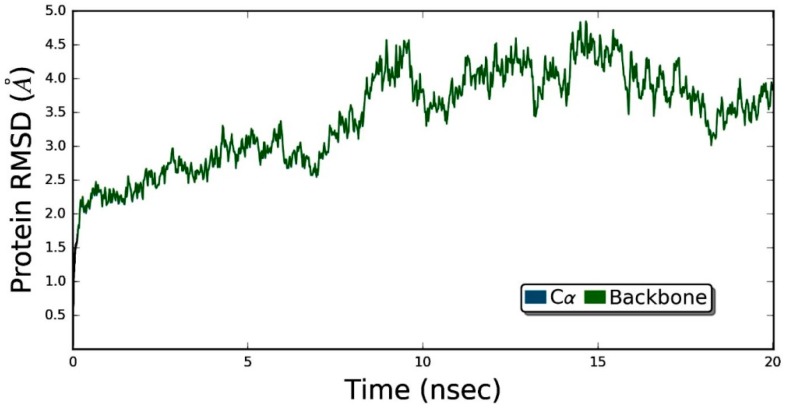
The root mean square deviation (RMSD) values for protein are shown on the y-axis during the simulation time of 20 nsec for ZER-integrin αvβ3 protein complex in the presence of TP5-iRGD peptide.

**Figure 9:**
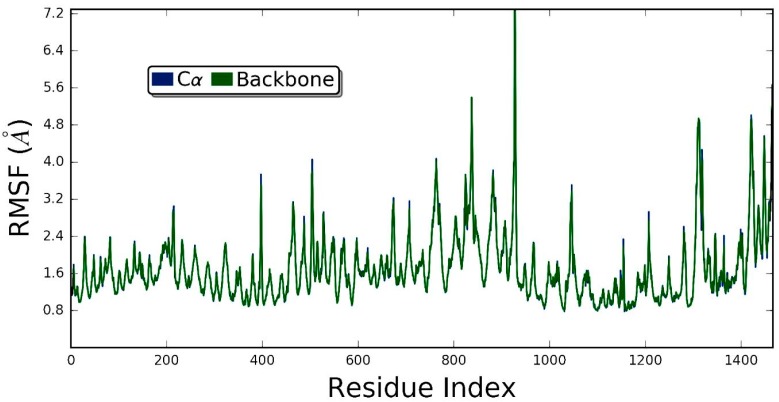
The root meansquare fluctuation (RMSF) of ZER-integrin αvβ3 protein complex in the presence of TP5-iRGD peptide during 20 ns molecular dynamics simulation.

Figure 8 demonstrates that the ZER did not disrupt the structural integrity of the integrin αvβ3 within the 20 ns simulation time period. The RMSD graph of the simulated complex (Figure 9) showed that the trajectory of the integrin αvβ3 protein was initiated by deviations caused by fluctuations of several amino acids in the beginning (the equilibration period) and slowly peaked up after 5 nsec time evolution and, after that, the protein remained quite stable, maintaining the RMSD below 5 Å. These results prove the protein’s stability in the dynamic environment.

Generally, RMSD is considered as a global measurement of protein motion, whereas root mean- square fluctuation (RMSF) is recognized as a local measurement that provides a higher resolution detail of residue fluctuations. Therefore, the flexibility of the ZER -integrin αvβ3 complex was further ascertained by measuring the RMSF values (Figure 9). The RMSF of protein has demonstrated flexibility within the range of 1.6 to 7.2 Å, showing that the binding of ZER stabilizes the affinity in the protein active site. In other words, no conformational and structural changes occur along the protein side chain during the simulated time.

**Figure 10:**
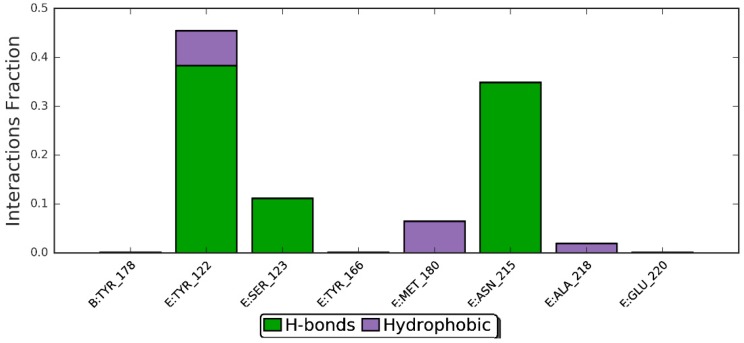
Per-residues analysis of the ZER in complex with integrin αvβ3 protein in the presence of TP5-iRGD peptide; the analysis was based throughout the 20 ns molecular dynamics simulations.

**Figure 11:**
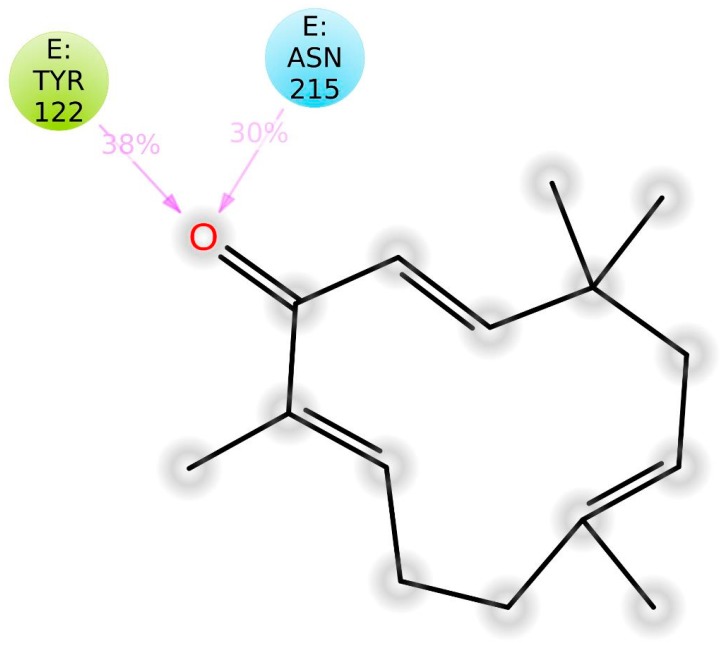
A representation of atomic interactions between ZER and integrin αvβ3 protein residues during 20 ns molecular dynamics simulation.

Protein–ligand interaction types were been monitored throughout the simulation, and are displayed in Figure 10. The bar chart is normalized over the course of the trajectory (i.e., 0.7 means the specific interaction was maintained 70% of the simulation time). Observed interactions can be categorized into twotypes: hydrogen bonds andhydrophobic contacts. Key residues involved in hydrogen bonding include Tyr-122, Ser-123, and Asn-215. However, Tyr-122 has also served in the hydrophobic interaction. In addition, Met-180 and Ala-218 also contributed through nonpolar contacts. Ligand–protein contacts observed during simulation of ZER are also shown in Figure 11.

## 4. Discussion

One of the crucial causes for the lack of efficacy of anticancer agents is poor penetrability across the biological membrane, which is considered as an obstacle in the treatment of tumors. In this study, we demonstrated that the in vitro anticancer activity of ZER against breast cancer cells could be enhanced upon co-administration with TP5-iRGD peptide by inducing apoptosis. Furthermore, in silico results of docking and molecular dynamics simulations rationalize the interaction of ZER with αvβ3integrin, which is known to be overly-expressed on both angiogenic vessels and tumor cells. It has been reported that in the field of cancer therapy, the overexpression of integrin receptors on the surface of various malignant tumour cells has been increasingly of interest [49]. Targeted delivery of therapeutic agents into tumors constitutes a major goal in cancer therapeutics. By augmenting the amount of a drug reaching tumor, the efficacy is improved while side effects are reduced [7]. Tumor tissues are characterized by distinctive pathological hallmarks such as an enormous volume of random vasculature with high permeability, a dense extracellular matrix, high interstitial fluid and osmotic pressure, the absence of lymphatic drainage, and an acidic and anoxic environment in the center of the tumor that is caused by poor elimination of metabolites owing to insufficient blood supply [50]. Consequently, antitumor agents are incapable of reaching deep into the tumor parenchyma, which leads to accumulation of drugs around the tumor blood vessels. This inconsistent distribution of drugs is regarded as one of the critical reasons for relapse and metastasis, impeding the development of suitable treatment regimen for cancer.

The results of the MTT cell viability assay indicated that ZER and ZER encapsulated in hydroxypropyl-β-cyclodextrin (ZER-HPβCD) inhibited the growth of MCF-7 breast cancer cells at 72 h treatment, with IC_50_ of 7.51 and 5.08 µg/mL, respectively, and inhibited the growth of MDA-MB-231 breast cancer cells, exhibiting IC_50_ of 14.96 and 12.18 µg/mL, respectively. Interestingly, ZER and TP5-iRGD peptide showed no significant cytotoxicity against Hs27 normal cells and the MTT assay of individual compounds showed high selectivity, with an IC_50_ greaterthan 100 and 1000 µg/mL, respectively, against Hs27 normal cells. Therefore, we considered both compounds to be safe, however, the co-administration was not tested against the normal cells and this could be one of the limitations of the study. Table 2 shows that ZER and ZER-HPβCD complex exhibit the highest percentage of inhibition (91.4 and 90.4%, respectively) against estrogen receptor positive breast cancer cells (MCF-7) when co-administered with 10× of TP5-iRGD peptide, however, the percentage of inhibition inversely reduced by increasing the ratio of peptide to 20× or 30×. On the other hand, there was no increased in the antiproliferative effect of ZER against the triple negative breast cancer cells (MDA-MB-231) with co-administration with the peptide even at 10× ratio, while ZER-HPβCD complex with TP5-iRGD showed slightly increased in the antiproliferative activity. This may indicate that the co-administration may interfere with a certain signalling pathway that is involved in the pathogenesis of estrogen receptor positive breast cancer cells, but not part of MDA-MB-231 cell proliferation.

Co-administration of TP5-iRGD peptide in MCF-7 cells reduced the IC_50_ of ZER from 7.51 µg/mL to 3.13 µg/mL, whereas IC_50_ of ZER-HPβCD complex was reduced from 5.08 µg/mL to 0.49 µg/mL. These results buttress the reports of other laboratories emphasising the cell penetration capabilities of TP5-iRGD peptide upon co-administration with various anticancer agents [2]. iRGD (CRGDKGPDC) is a tumour-penetrating peptide, capable of homing and penetrating into tumour cells [35]. It binds to αvβ3 integrin, which are highly and specifically expressed within tumour endothelium, but not in normal tissues, thus enhancing the therapeutic effect of antitumor drugs on suppressing tumour growth and/or metastasis. After binding to αvβ3 integrin, the iRGD peptide is proteolytically cleaved to produce CRGDK fragment, which favours binding to neuropilin-1 receptor, hence easing the penetration of drugs into the tumour [51].

Zerumbone, a sesquiterpene compound containing two conjugated and one isolated double bonds and α, β-unsaturated carbonyl group in the 11-membered ring structure (Figure 7), exerted potent anticancer activities in breast cancer cells upon co-administration with TP5-iRGD peptide. TP5-iRGD increased vascular and tissue permeability, allowing co-administered ZER to penetrate into extravascular tumor tissue. Interestingly, exploitation of the beneficial effects of TP5-iRGD did not require ZER to be chemically conjugated to the peptide.

The results of the AO/PI staining under fluorescence microscopy showed evidence of early apoptosis after 72 h from the co-administration of ZER or ZER -HPβCD with TP5-iRGD peptide in MCF-7 breast cancer cells. Hence, it could be that zerumbone-TP5-iRGD complex induces apoptosis in breast cancer cells via targeting αvβ3 integrin.

Advances in computer hardware and force fields development enabled molecular dynamics (MD) simulation as a powerful computational tool that can shed light on the specific molecular interactions between protein and inhibitor at the atomic level [52,53]. Atomistic molecular dynamics simulations were performed on docked ligand–protein complex, in order to understand inhibitor specificity, the active site loop, and key residues within the binding pocket. Coordinates of the lowest energy configuration were subjected to MD simulation using Desmond [44]. Molecular dynamics simulation was performed in conditions that closely replicate physiological environment. The ligand–protein complexes were immersed in water and the system was neutralized with Na^+^/Cl^−^ counter-ions using a salt of concentration 0.15 M.

The root mean square deviation (RMSD) of a protein, measured as a function of time, is regarded as an indicator of the convergence in protein structure change over the course of a simulation [54].

## 5. Conclusions

In conclusion, the cytotoxicity of zerumbone against estrogen receptor positive breast cancer (MCF-7) cellswas significantly increased, and the potencyimproved through co-administration with TP5-iRGD peptide. The fluorescence microscopy after 72 h of co-administration indicated that cancer cells undergo early and late apoptosis. Molecular modelling provides novel insights into the ZER interaction with integrin αvβ3 in the presence of TP5-iRGD, and this could explain the antitumor activity of ZER when co-administered with TP5-iRGDpeptide. Therefore, these promisingresults represent a good strategy to enhance the antitumor activity of ZER in fightingbreast cancerwhen co-administer with safe non-toxic cell penetrating peptide such as TP5-iRGD. Further investigations including proteomic and molecular studies are needed to understand the underlying mechanism of action.

## Figures and Tables

**Table 1:** Inhibitory concentration (IC)_50_ of zerumbone (ZER), ZER-hydroxypropyl-β-cyclodextrin(HPβCD) and TP5-iRGD peptide.

Cell Lines	ZER			ZER-HPβCD			TP5-iRGD Peptide	
	24 h IC_50_ (µg/mL)	48 h IC_50_ (µg/mL)	72 h IC_50_ (µg/mL)	24 h IC_50_ (µg/mL)	48 h IC_50_ (µg/mL)	72 h IC_50_ (µg/mL)	48 h IC_50_ (µg/mL)	72 h IC_50_ (µg/mL)
**MCF7**	17.36 ± 2.3	9.06 ± 1.0	7.51 ± 0.2	20.81 ± 9.3	4.43 ± 0.2	5.08 ± 0.2	>1000	>1000
**MDA-MB-231**	50.93 ± 3.0	23.44 ± 10.0	14.96 ± 1.5	40.73 ± 1.9	21.1 ± 6.7	12.18 ± 0.7	>1000	>1000
**Hs27**	>100	>100	>100	42.99 ± 2.3	46.14 ± 2.4	21.45 ± 1.3	>1000	>1000

Data are presented as mean ± SD; ZER: zerumbone, ZER-HPβCD: zerumbone encapsulated in hydroxypropyl-β-cyclodextrin.

**Table 2:** Of cell growth inhibition of MCF-7 and MDA-MB-231 breast cancer cells after 72 h of co-administration of ZER or ZER-HPβCD with TP5-iRGD peptide.

Co-administration Ratio	MCF-7 (% of Inhibition)	MDA-MB-231 (% of Inhibition)		
Compound (IC_50_): TP5-iRGD	ZER	ZER-HPβCD	ZER	ZER-HPβCD
**1:10 X**	91.4 ± 3.0	90.4 ± 4.2	58.1 ± 8.8	41.5 ± 20.4
**1:20 X**	67.6 ± 3.9	85.8 ± 7.0	44.0 ± 8.6	72.8 ± 2.3
**1:30 X**	72.1 ± 8.6	68.5 ± 13.2	48.3 ± 5.4	79.1 ± 1.8

Data are presented as mean ± SD; ZER: zerumbone, ZER-HPβCD: zerumbone encapsulated in hydroxypropyl-β-cyclodextrin.

**Table 3:** The co-administration of ZER and ZER-HPβCD with TP5-iRGD peptide against MCF-7 cells after 72 h treatment. The co-administration of ZER or ZER-HPβCD with TP5-iRGD peptide at a ratio 1:10 in MCF-7 cells reduced the IC_50_ of ZER from 7.51 ± 0.2 µg/mL to 3.13 ± 0.7 µg/mL and reduced the IC_50_ of ZER-HPβCD from 5.08 ± 0.2 µg/mL to 0.49 ± 0.004 µg/mL after 72 h treatment.

Co-Administration	MCF-7–IC_50_ (µg/mL)
**ZER with TP5-iRGD**	3.13 ± 0.7
**ZER-HPβCDwith TP5-iRGD**	0.49 ± 0.004

Data are presented as mean ± SD; ZER: zerumbone, ZER-HPβCD: zerumbone encapsulated in hydroxypropyl-β-cyclodextrin.

**Table 4:** Obtained afterdocking of ZER with integrin αvβ3.

Parameters	ZER	ZER in the Presence of TP5-iRGD
Binding Energy, ΔGb (kcal/mol)	−6.77	−8.13
Predicted Ki (µM)	10.97	1.10
RMSD	4.39	4.16
Residues involved in hydrophobic interactions	Met-180(B), Arg-214(B)	Tyr-122(B), Tyr-166(B), Tyr-178(A), Arg-214(B), Arg-216(B), Ala-218(B)
Residues involved in hydrophilic interactions	Nil	Nil

RMSD: root mean square deviation.
